# The Role of Siglec-1 and SR-BI Interaction in the Phagocytosis of Oxidized Low Density Lipoprotein by Macrophages

**DOI:** 10.1371/journal.pone.0058831

**Published:** 2013-03-08

**Authors:** Yi-song Xiong, Juan Yu, Chang Li, Lin Zhu, Li-juan Wu, Ren-qian Zhong

**Affiliations:** 1 Department of Laboratory Medicine, Chengdu Military General Hospital, Chengdu, China; 2 Department of Laboratory Medicine, Changzheng Hospital, Second Military Medical University & Clinical Immunology Center of PLA, Shanghai, China; 3 Department of Cardiology, Changzheng Hospital, Second Military Medical University, Shanghai, China; UAE University, Faculty of Medicine & Health Sciences, United Arab Emirates

## Abstract

**Background:**

Macrophages play a proatherosclerotic role in atherosclerosis via oxLDL uptake. As an adhesion molecular of I-type lectins, Siglec-1 is highly expressed on circulating monocytes and plaque macrophages of atherosclerotic patients, but the exact role of Siglec-1 has not been elucidated.

**Methods:**

In this study, oxLDL was used to stimulate Siglec-1 and some oxLDL receptors (SR-BI, CD64, CD32B, LOX-1 and TLR-4) expression on bone marrow-derived macrophages, whereas small interfering RNA was used to down-regulate Siglec-1. Meanwhile, an ELISA-based assay for Siglec-1-oxLDL interaction was performed, and co-immunoprecipitation (co-IP) and laser scanning confocal microscopy (LSCM) were used to determine the role of Siglec-1 in oxLDL uptake by macrophages.

**Results:**

We found that oxLDL could up-regulate the expression of various potential oxLDL receptors, including Siglec-1, in a dose-dependent manner. Moreover, down-regulation of Siglec-1 could attenuate oxLDL uptake by Oil red O staining. LSCM revealed that Siglec-1 and CD64/SR-BI may colocalize on oxLDL-stimulated macrophage surface, whereas co-IP showed that Siglec-1 and SR-BI can be immunoprecipitated by each other. However, no direct interaction between Siglec-1 and oxLDL was found in the *in vitro* protein interaction system.

**Conclusions:**

Thus, Siglec-1 can interact with SR-BI in the phagocytosis of oxLDL by macrophages, rather than act as an independent receptor for oxLDL.

## Introduction

Reduction of plasma level of oxidized LDL (oxLDL) may have a protective role in the initiation and progression of atherosclerosis (AS). Ishigaki et al [1] found that lowering oxLDL alone, rather than total LDL, affected atherogenesis in apolipoprotein E-deficient mice. Laukkanen et al [2] found that adenovirus-mediated gene transfer of a secreted form of human macrophage scavenger receptor (SR) inhibited modified LDL degradation and foam-cell formation in macrophages. Schiopu et al [3] found that recombinant antibodies to an oxLDL epitope can induce rapid and substantial regression of atherosclerosis in mice, possibly by stimulating lipid efflux and inhibiting macrophage recruitment. Binder et al [4] found that pneumococcal vaccination can decrease atherosclerotic lesion formation via molecular mimicry between *streptococcus pneumoniae* and oxLDL. These results together suggest that oxLDL has a major atherogenic role, and oxLDL removal might prevent the development of atherosclerosis, at least partly, due to inhibition of oxLDL incorporation into macrophages.

Many receptors for oxLDL have been identified, most of which belong to the SR family and FcγR family [5]. Siglec-1 is originally found as a lectin-like adhesion molecule of 185-kDa expressed on specific macrophage subpopulations. Siglec-1 can mediate both sialic-acid-dependent and sialic-acid-independent interactions with cells of the immune system [6]. Siglec-1(+) macrophages can internalize lipid antigen and process and present it to iNKT cells, resulting in T cells proliferation and activation [7]. Furthermore, Siglec-1 on macrophage can serve as receptor for some virus and facilitate virus infection of host cells [8], [9]. However, whether Siglec-1 plays a role in macrophage uptake of lipoprotein is still unclear.

Accordingly, we desire to explore the role of Siglec-1 in macrophage oxLDL uptake. Firstly, oxLDL 100 µg/ml was used to stimulate the expression of Siglec-1 and some validated oxLDL receptors on macrophages; Secondly, small interfering RNA (siRNA) was used to down-regulate the expression of Siglec-1 and the capacity of oxLDL internalization by macrophages was observed; Thirdly, an ELISA-based assay for Siglec-1-oxLDL interaction was performed, and co-immunoprecipitation and LSCM were used to determine the role of Siglec-1 in oxLDL uptake.

## Materials and Methods

Detailed methods can be found in File S1.

### FACS

All animals received humane care and protocols for animal experiments were approved by the institutional animal use committee of the Second Military Medical University. Mouse bone marrow-derived macrophages (BMMs) were stimulated with different concentration of oxLDL (0, 12.5, 25, 50, 100 µg/ml) for 48 h and harvested by 0.25% trypsin-1 mM EDTA solution (Gibco). 2×10^5^ cells in 100 µl staining buffer (PBS +0.5% BSA +0.05% sodium azide) were firstly Fc-blocked with 2 µg of mouse IgG for 15 minutes at room temperature and subsequently incubated with antibody for Siglec-1, CD64, CD32B, TLR-4, LOX-1 or SR-BI at a concentration of 10 µg/ml for 1 h. After wash, cells were resuspended in 100 µl staining buffer, stained with appropriate DyLight™ conjugated secondary antibody at a concentration of 5 µg/ml for 30 min. And then washed and resuspended in 500 µl PBS. Cells were analyzed by FC500 flow cytometer and CXP Analysis Softwares (Beckman Coulter). Appropriate isotype-matched control antibodies were used in parallel.

### Semi-quantitative RT-PCR

PCR analysis was performed as described previously [10]. Briefly, total RNA was extracted by using RNeasy mini kit (Qiagen, Hilden, Germany). To avoid genomic DNA contamination, DNA degradation was performed by using RQ1 RNase-Free DNase (Promega, Madison, WI). cDNA was synthesized by using the SuperScript III First-Strand Synthesis kit (Invitrogen) with oligo dT primers. Primers were designed with the Primer Express software, version 3.0 (Applied Biosystems, Foster City, CA) and verified to generate a single product specific to target genes by BLAST algorithm (http://www.ncbi.nlm.nih.gov/blast/). Primers were as follow: mouse Siglec-1 (**NM_011426.3**), sense-primer, 5′-CCTGGTGTGCAGTGTACAAAGTG-3′, antisense-primer, 5′-CCGCGCCTTGTAGGGTAGA-3′, amplicon size 89 bp; mouse GAPDH (**NM_008084.2**), sense-primer, 5′-TGGCCTCCAAGGAGTAAGAAAC-3′, antisense-primer, 5′-GGGATAGGGCCTCTCTTGCT-3′, amplicon size 72 bp. Real-time PCR reactions were performed by using the ABI 7000 System with SYBR® Green PCR Master Mix (Applied Biosystems). Specificity of the products was confirmed by melting curve analysis and gel electrophoresis. As a control for cross contamination samples consisting of distilled water were also subjected to the isolation procedures and the extracts were tested with all assays. Cycle Threshold (Ct) values were calculated after confirming similar amplification efficiencies of target gene and endogenous control. Results were analyzed using ΔΔCt method [11].

### Oil Red O Staining

BMMs were plated on chamber slides and stimulated with oxLDL 100 µg/ml for 48 h. After incubation, the cells were washed with PBS, fixed with 4% paraformaldehyde for 10 minutes, and stained with a saturated concentration of oil red O in 60% isopropanol for 1 h at 55°C. After washed with PBS, slides were mounted with glycerogelatin and photographed with a Leica DM LB microscope (Leica Microsystems) equipped with an Olympus DP70 CCD camera (Olympus). For quantitation of lipid accumulation in cells, 1×10^5^ cells were washed, fixed and stained as above. And then cells were washed with 60% isopropanol for 5 s and incorporated stain was eluted with 300 µl 60% isopropanol and the optical density (OD) of the solution at 510 nm was measured [12], [13].

### Siglec-1-oxLDL Interaction Assay by ELISA

ELISA for protein interaction was performed as described previously [14], [15]. Briefly, recombinant human or mouse Siglec-1 (0.2 µg∼0.5 µg/100 µl, 5197-SL, 5610-SL, both from R&D systems) or BSA (0.2 µg/100 µl, as a negative control) was immobilized to each well of 96-well ELISA plates (high-binding, Corning Costar 9018) by incubation overnight (16–20 h) at 4°C in PBS. After 2 washes with wash buffer (PBS+0.1% Tween 20), the plates were blocked with 300 µl blocking buffer (3% BSA/PBS) at 4°C for 12 h. After 2 washes with wash buffer, different concentration of oxLDL (1.53 ng/ml∼200 µg/ml) in the 1×Assay Diluent (eBioscience) was added to each well, and incubated at 4°C overnight. In some cases, *Vibrio cholerae* sialidase (50 mU/ml, Sigma) was used to treat rh-Siglec-1 and oxLDL for 1 hour at 37°C before adding them to the well [16]. The plates were then washed and incubated with rabbit polyclonal to oxLDL (1∶1000, Abcam or Chemicon) in Assay Diluent at 4°C overnight. After wash, plates were incubated with HRP conjugated goat anti-rabbit (1∶5000, Jackson ImmunoResearch) in Assay Diluent for 2 h at room temperature. After 7 washes with wash buffer, peroxidase activity was determined with a TMB Substrate Solution (eBioscience) and absorbance at 450 nm was measured on an automatic plate reader (Bio-Rad Laboratories). For standard curve, 2-fold serial diluted oxLDL (1.53 ng/ml∼200 µg/ml) was immobilized directly to 96-well ELISA plates and blocked, subsequently primary and secondary antibody were used and peroxidase activity was determined as above. For negative control, the primary antibody was substituted with normal rabbit serum at the same dilution. A known oxLDL receptor CD36 was used as positive control to validate the ELISA system.

### Laser Scanning Confocal Microscopy (LSCM)

BMMs were plated on 6-well plate with sterile coverslip, stimulated with oxLDL 100 µg/ml for 48 h. Culture medium was discarded and cells were washed with PBS twice and fixed with 4% paraformaldehyde for 10 minutes. After Fc-blocked with blocking buffer (PBS +0.5% BSA +10%FBS) for 40 min, cells were incubated simultaneously with anti-Siglec-1 and one of the following antibody: CD36, SR-A, CD64, CD32B, TLR-4, LOX-1 or SR-BI at a concentration of 20 µg/ml diluted in blocking buffer overnight at 4°C in a humidified container. Appropriate isotype-matched IgG or normal serum was used in parallel as control. After washed with PBS, cells were incubated successively with appropriate DyLight™ conjugated secondary antibody at a concentration of 10 µg/ml diluted in blocking buffer for 30 min in the dark, with wash steps between each incubation. Then coverslips were mounted with Prolong® Gold Antifade Reagent (Invitrogen) and analyzed using a Leica TCS SP2 confocal microscope and TCSNTV software.

### Co-immunoprecipitation (Co-IP) and Immunoblotting (IB)

BMMs were stimulated with oxLDL (100 µg/ml), PMA(20 ng/ml) or LPS(0.1 µg/ml) for 48 h, washed 3 times with ice-cold PBS and lyzed and cell membrane protein was extracted by using transmembrane protein extraction kit (Novagen) with Extraction Buffer 2A. Protein concentrations were determined using the BCA protein assay kit (Pierce). A Pierce® Classic IP Kit was used for Co-IP. Briefly, 3 µg of rat anti-Siglec-1 antibody (3D6.112) or rabbit polyclonal to SR-BI (Thermo Scientific) was mixed with 0.5 mg extracted membrane protein in a microcentrifuge tube overnight at 4°C to form immune complex. Then the immune complex were captured and eluted according to the manufacturer’s instructions.

Twenty micrograms per lane of immunoprecipitated protein were mixed with one-fifth volume of sample buffer and boiled for 5 min before being loaded onto a 5% stacking/12% resolving gel (5% stacking/8% resolving gel for Siglec-1) for electrophoresis. Separated proteins were transferred to PVDF membrane (Millipore, Billerica, MA) using an Mini Trans-Blot® Electrophoretic Transfer Cell (Bio-Rad) with transfer buffer (25 mmol/L Tris base, 192 mmol/L glycine, 20% methanol, PH 8.3) at 300 mA for 1 h in ice bath. Blots were rinsed in water, washed in TBST (20 mmol/L Tris-HCl (pH 7.6)/137 mmol/L NaCl/0.1% Tween 20) and incubated 1.5 h in blocking solution (5% w/v nonfat dry milk in TBST). Blots were incubated overnight at 4°C in blocking solution containing primary antibodies for Siglec-1, CD36, SR-A, CD64, CD32B, TLR-4, SR-BI, LOX-1, caveolin-1 or Na/K ATPase (concentrations between 0.5∼4 µg/ml, see Table S1). The next day, membranes were washed 5 times, 5 min each, in TBST prior to 1 h incubation at room temperature with appropriate HRP-conjugated secondary antibody (Jackson ImmunoResearch, 1∶5000∼1∶10000 dilution) diluted in blocking solution. After membranes were washed for 7 times, 5 min each in TBST, signal was detected by using ECL chemiluminescent substrate (Millipore) and recorded by X-ray films (Kodak, Rochester, NY).

### Statistical Analysis

Data were shown as mean±SD. Statistical analysis was performed using the SPSS V.15.0 for Windows software (SPSS Incorporated). Normally distributed data were analyzed using one-way ANOVA followed by Student-Newman-Keuls post-hoc test. For non-normally distributed data, significant analyses between more than two groups was performed using Kruskal–Wallis H test instead, significant difference between two groups was analyzed using Mann–Whitney U test. The significance level was set at *P*<0.05.

## Results

### OxLDL can Stimulate the Expression of Various Receptors on Macrophages

**Figure 1:**
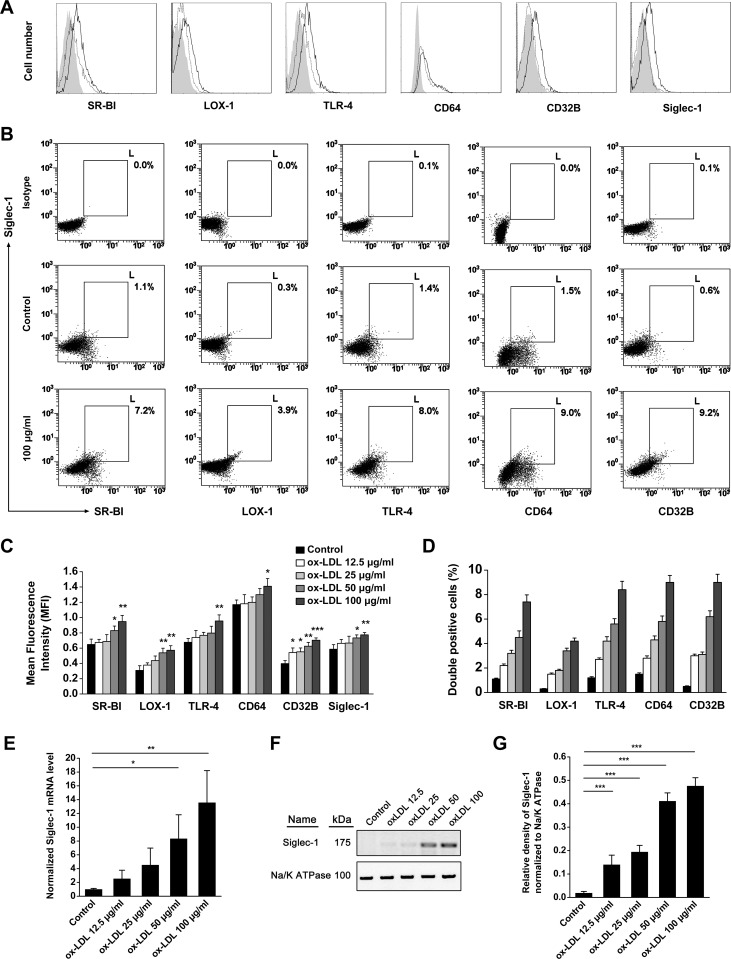
OxLDL can up-regulate many receptors, including Siglec-1. BMMs were stimulated with different concentration of oxLDL (0, 12.5, 25, 50, 100 µg/ml) for 48 h and harvested for FACS, QRT-PCR and Western blot analysis. (A), Single parameter histogram of different receptors expression, abscissa represent cell number and ordinate represent fluorescence intensity. Shadow areas indicate isotype control, dotted lines indicate oxLDL 0 µg/ml and solid lines indicate oxLDL 100 µg/ml. (B), Double-parameter analysis of Siglec-1 and other oxLDL receptors. (C), Statistical analysis of panel A (n = 6). oxLDL can stimulate these receptors expression in a dose-dependent manner. *p<0.05,**p<0.01,***p<0.001 vs. their own control groups. (D), Statistical analysis of panel B, legend as in panel C, all treatment groups have *p* values <0.001 vs. their own control groups (n = 6). (E), QRT-PCR detection of Siglec-1 expression by the stimulation of different concentrations of oxLDL. *p<0.05,**p<0.01. (F–G), Western blot detection of Siglec-1 expression by oxLDL stimulation. ***p<0.001.

As shown in fig. 1, oxLDL can stimulate the expression of SR-BI, LOX-1, TLR-4, CD64, CD32B and Siglec-1 on BMMs in a dose-dependent manner (fig. 1A, 1C). Among these receptors, SR-BI, LOX-1, CD64 and CD32B are well defined oxLDL receptors, whereas TLR-4 is recently realized to have important role in oxLDL uptake [17]. OxLDL can also dose-dependently stimulate Siglec-1 mRNA and protein expression on macrophage (fig. 1E–G), which indicates that Siglec-1 may act as a novel receptor for oxLDL. Moreover, by the stimulation of oxLDL, Siglec-1 and other receptors can overexpress simultaneously on one cell (fig. 1B, 1D), which implies Siglec-1 may work synergetically with other receptors in oxLDL uptake. Another two major oxLDL receptors, SR-A and CD36, can also be up-regulated by oxLDL stimulation (data not shown).

### Siglec-1 Knockdown can Inhibit BMMs Internalization of oxLDL

**Figure 2:**
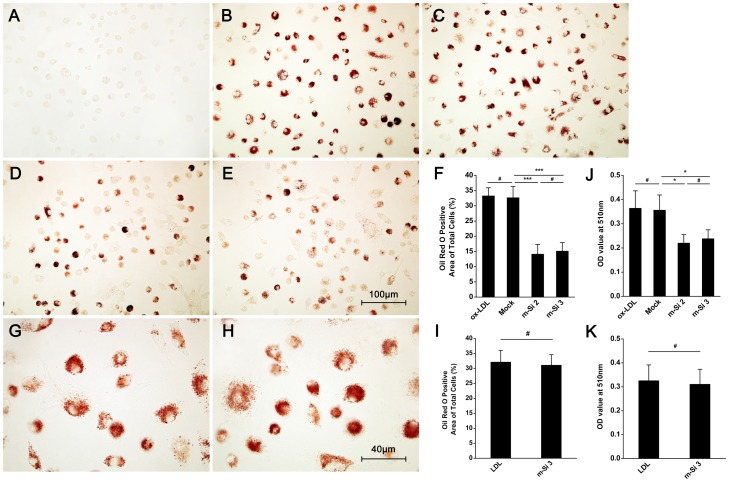
Siglec-1 knockdown can attenuate oxLDL uptake by macrophages. BMMs were cultured for 6 days and transfected with mock siRNA (C) or siRNA targeting Siglec-1 (D, E, H), 24 h later, oxLDL 100 µg/ml (B∼E) or LDL 100 µg/ml (G∼H) was added and stimulated for 48 h. Then Oil red O staining was performed (A∼E, G∼H). Red particles in the cytoplasm indicated Oil red O positive lipid. A, control; B, oxLDL 100 µg/ml; C, mock transfection; D, m-si 2; E, m-si 3. Magnification ×400 (A–E). (F), Statistical analysis of A–E, randomly 20 highpower fields were selected in each group. #*p*>0.05, ***p<0.001. (G), native LDL 100 µg/ml was used as control. (H), LDL 100 µg/ml+m-si 3. Magnification ×1000 (G–H). (I), Statistical analysis of G–H. #*p*>0.05. (J), Quantitation of Oil red O staining in B–E with a spectrophotometric method. #*p*>0.05, *p<0.05. (K), Quantitation of Oil red O staining in G–H. #*p*>0.05.

As shown in fig. 2, oxLDL can be internalized by normal BMMs, with oil red O positive red particles in the cytoplasma of every cell (fig. 2B, 2C). However, when Siglec-1 was blocked by siRNA (fig. S1), the capacity of oxLDL uptake by macrophages was significantly reduced, with fewer positive cells and fewer red particles in each cell (fig. 2D–F). Most importantly, other major oxLDL receptors including SR-A, SR-BI, CD36 and CD64 were not affected by Siglec-1 inhibition (fig. S2). When Siglec-1 was inhibited by siRNA, the capacity of native LDL internalization by BMMs was not affected (fig. 2G–I). Quantitation of intracellular lipid accumulation with a colorimetric method showed similar results of the image-based analysis (fig. 2J, 2K). Similar results were also found in RAW264.7 cells (data not shown).

### No Direct Interaction between Siglec-1 and oxLDL was found by ELISA

**Figure 3:**
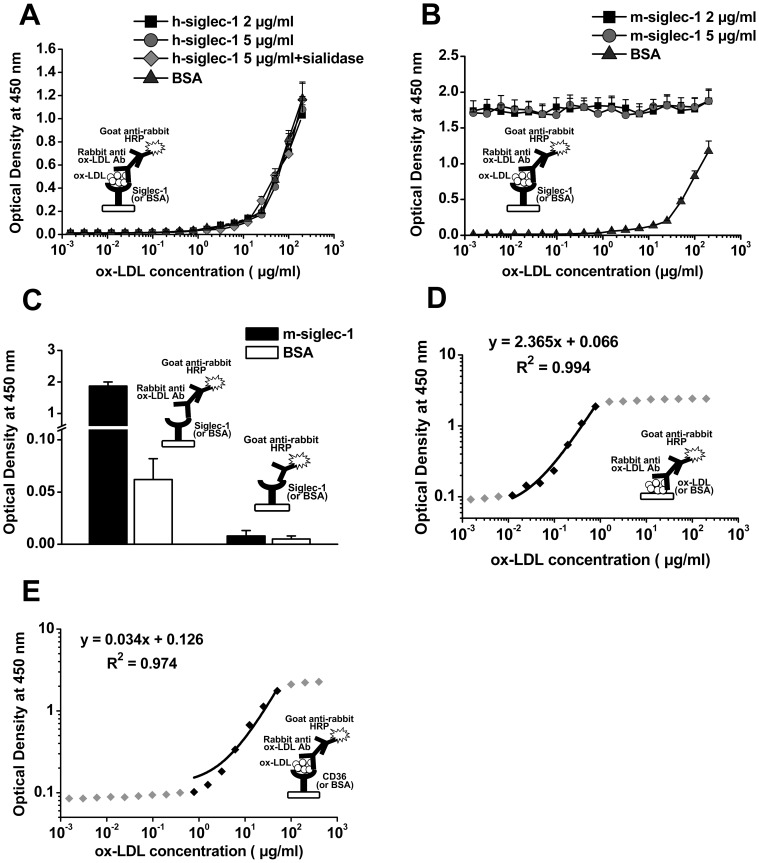
No direct interaction between h-Siglec-1/m-Siglec-1 and oxLDL was observed by ELISA. Different concentrations of recombinant human (A) or mouse (B) Siglec-1 or BSA were immobilized to 96-well ELISA plates, blocked with BSA and incubated with serial diluted oxLDL (1.53 ng/ml∼200 µg/ml). Then the binding of oxLDL to Siglec-1 was determined. At high concentration (50∼200 µg/ml), oxLDL can nonspecifically adsorb to incompletely blocked plates. No interaction between h-Siglec-1 (A) and oxLDL (in neither native form nor desialylated form) was found as compared with BSA. (B), m-Siglec-1 can strongly bind oxLDL, even though in very low concentration. However, the pseudo-binding was result from interaction between m-Siglec-1 and anti-oxLDL antibody (C), Data were expressed as mean±SD and n = 8 for each group. (D), serial diluted oxLDL (1.53 ng/ml∼200 µg/ml) were directly immobilized to ELISA plates and linear range of this ELISA-based method for oxLDL detection was determined as 0.01∼1 µg/ml. (E), A known oxLDL receptor CD36 was used as positive control to validate the ELISA system. CD36 can bind oxLDL with the linear range of 0.78-50 µg/ml.

*In vitro* studies have demonstrated that LOX-1, SR-PSOX, and SREC bind modified LDL with dissociation constants in the range of 3–36 µg/ml, comparable with that of SR-A [18]. In this study, we introduced an ELISA-based protein interaction method to determine whether Siglec-1 can bind directly to oxLDL [14]. As shown in fig. 3, the ELISA-based method can detect oxLDL with a linear range of 0.01∼1 µg/ml (fig. 3D). Unfortunately, neither h-Siglec-1 nor m-Siglec-1 can interact directly with oxLDL in their native form and desialylated form, as compared with BSA (fig. 3A, 3B). However, m-Siglec-1 can interact with rabbit anti-oxLDL antibody (fig. 3C), resulting in a pseudo-positive signal in mSiglec-1-oxLDL interaction (fig. 3B). Mouse Siglec-1 can also react with many other primary antibodies (millipore anti-oxLDL, anti-SR-BI, data not shown), partly because of the Fc region of the recombinant protein. As a positive control, CD36 can bind oxLDL with the linear range of 0.78-50 µg/ml (fig. 3E). To eliminate the random error of experiments, Siglec-1 and anti-oxLDL were from two different sources, oxLDL were from 5 different batches, and all experiments were done for at least 8 times independently.

### Colocalization of Siglec-1 and CD64 or SR-BI on Macrophage Surface

**Figure 4:**
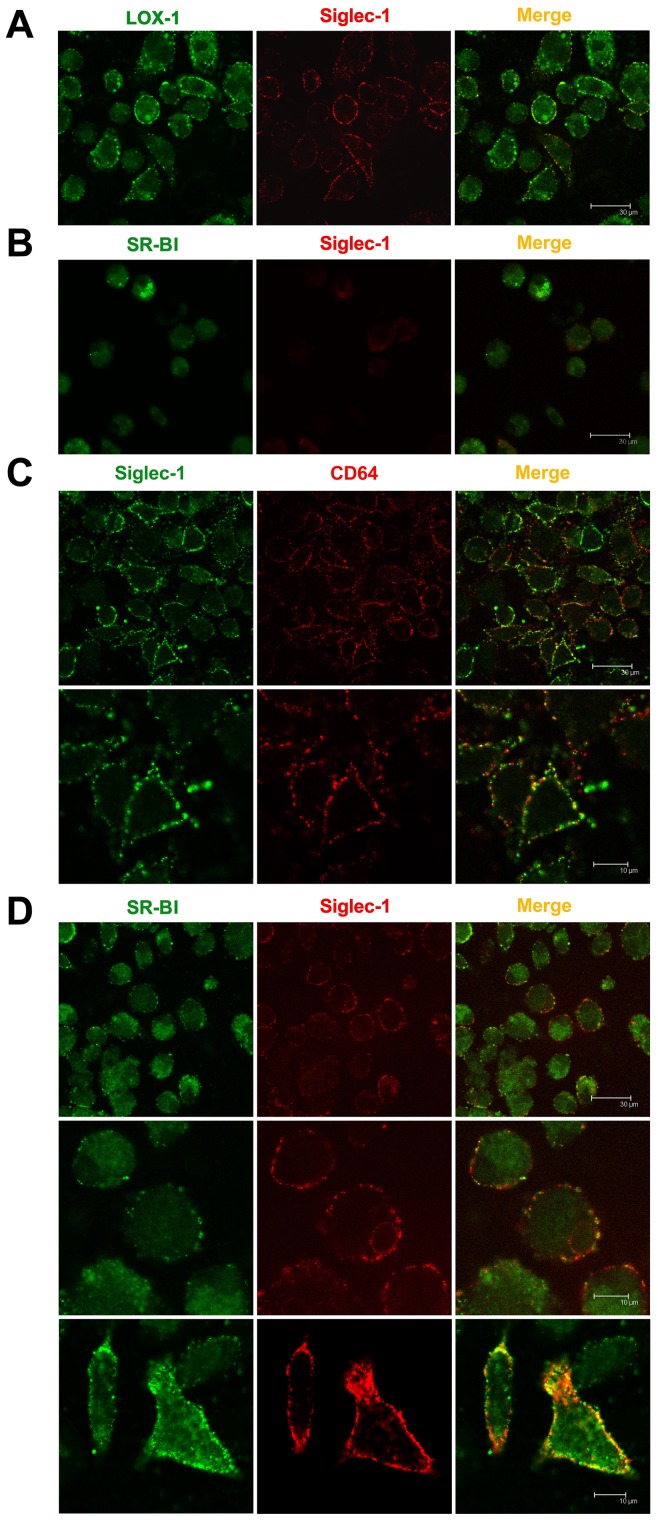
Colocalization of Siglec-1 and CD64/SR-BI on macrophage surface. BMMs were plated on 6-well plate with sterile coverslip, stimulated with oxLDL 100 µg/ml (A,C,D) or culture medium(B) for 48 h and cells were washed, fixed with 4% paraformaldehyde, and immunostaining simultaneously with anti-Siglec-1 and anti-CD64/anti-SR-BI/anti-LOX-1. Then cells were mounted and subject to LSCM analysis. Both CD64 (C) and SR-BI (D) may have a colocalization with Siglec-1 on cell surface as shown in merged graphs (yellow particles). But no colocalize of Siglec-1 with LOX-1 was found (A). When cells were not stimulated with oxLDL, the expression of Siglec-1 and SR-BI on cell surface was slight and colocalization of Siglec-1 and SR-BI was not found (B). Magnification ×400 (except for lower panel of C and D, ×1000).

By the stimulation of oxLDL, Siglec-1 and CD64 (fig. 4C) or SR-BI (fig. 4D) can be colocalized on BMMs surface, which indicated that the two receptors may work synergetically with each other in oxLDL uptake. Similar results were also found in RAW264.7 cells. We also tested other receptors including CD36, SR-A, CD32B, TLR-4 (data not shown) and LOX-1 (fig. 4A). However, no colocalization between Siglec-1 and other receptors were found except for CD64 and SR-BI. When cells were not stimulated with oxLDL, the expression of Siglec-1 and SR-BI on cell surface was slight and colocalization of Siglec-1 and SR-BI was not found (fig. 4B), which imply that stimulation with oxLDL is needed to have Siglec-1 co-localize with SR-BI.

### Siglec-1 and SR-BI can be Immunoprecipitated by Each Other

**Figure 5:**
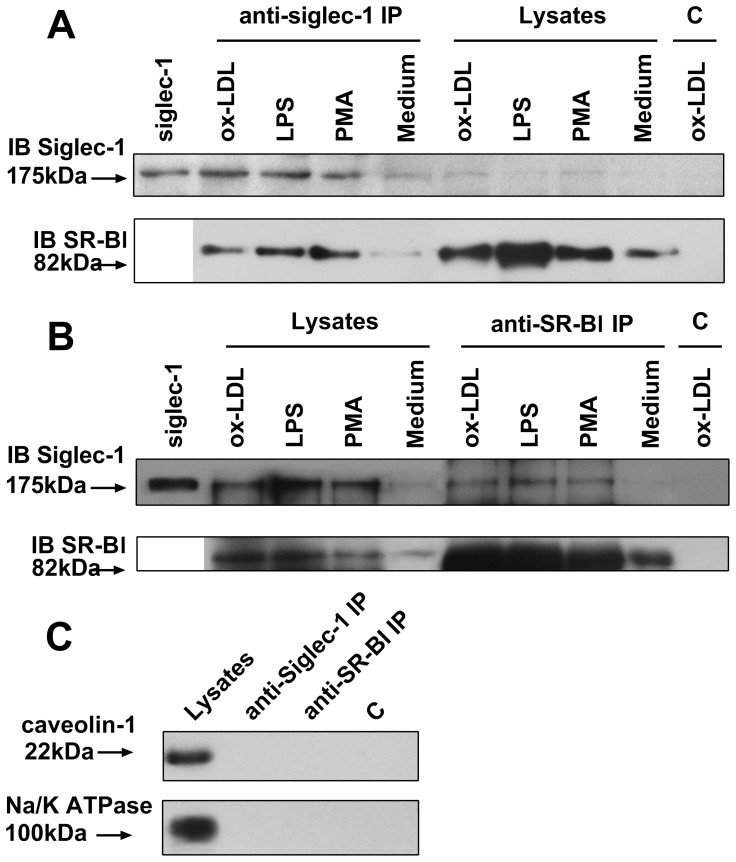
Siglec-1 and SR-BI can be immunoprecipitated by each other. (A,B), BMMs were stimulated with oxLDL (100 µg/ml), LPS (0.1 µg/ml), PMA (20 ng/ml) or culture medium (Medium) for 48 h and lyzed for cell membrane protein extraction. Then rat anti-Siglec-1 antibody or rabbit polyclonal to SR-BI was mixed with extracted membrane protein overnight to form immune complex. The immune complex were captured and eluted, and immunoblotting for Siglec-1/SR-BI was performed. Siglec-1 and SR-BI can be immunoprecipitated by each other. IP, immunoprecipitation; IB, immunoblotting; recombinant mouse Siglec-1 0.75 µg was used as positive control; Lysates indicates total cell membrane proteins (20 µg) were directly immunoblotted as a control (input); “C” indicates cells were immunoprecipitated with control IgG. (C), To exclude the possibility that Siglec-1/SR-BI was immunoprecipitated with the entire caveolae compartment, cells were stimulated with oxLDL 100 µg/ml, lysed and caveolin-1 in cell lysates and IP products was detected.

In this study, we used protein kinase C activator PMA [19], TLR-4 agonist LPS [20] and oxLDL [21] to up-regulate SR and other oxLDL receptors. Then cell membrane protein was extracted and co-IP was performed. As shown in fig. 5, Siglec-1 and SR-BI can be immunoprecipitated by each other in all the stimulated conditions (fig. 5A, 5B). More importantly, Siglec-1-SR-BI interactions also occur in the nonstimulated state (medium treated), although the signal is weak. However, CD36, SR-A, CD64, LOX-1, CD32B and TLR-4 cannot be immunoprecipitated by Siglec-1 and vice versa (data not shown). We also detected the expression of caveolin-1, the principal structural component of caveolae, by western blot in the immunoprecipitated product of anti-Siglec-1/anti-SR-BI. However, no caveolin-1 signal was detected (fig. 5C). This may exclude the possibility that Siglec-1/SR-BI was immunoprecipitated with the entire caveolae compartment.

## Discussion

LDL and oxLDL have different receptors. LDL is mainly recognized by LDL-R, whereas the oxidized form, with modified surface structure, cannot be recognized by LDL-R any more but by other receptors such as scavenger receptor (SR). Most importantly, the internalization of oxLDL is not regulated by the intracellular cholesterol level [22]. Many receptors for oxLDL has been identified to date, most of which belong to the SR family, including 1) SR-A I/II and MARCO (SR-A class) 2) SR-BI and CD36 (SR-B class), 3) CD68 (SR-D class), 4) lectin-like oxidized LDL receptor (LOX-1, SR-E class), 5) scavenger receptor expressed by endothelial cells (SREC, SR-F class), and 6) scavenger receptor for phosphotidylserine and oxidized lipoprotein (SR-PSOX/CXCL16, SR-G class) [5]. In addition to lipoproteins uptake, SR also plays an important role in pathogen recognition, including hepatitis C virus, whole bacteria or its components, such as LPS or lipoteichoic acid. OxLDL can also bond to FCγR [23] via oxLDL/beta2GPI-anti-oxLDL/beta2GPI complex, which has an important role in autoimmune diseases such as systemic lupus erythematosus and antiphospholipid syndrome [24].

As a member of Siglecs, Siglec-1 lacks tyrosine-based signaling motifs and its cytoplasmic tail is poorly conserved, which suggests a primary role as a binding partner in cell-cell interactions, rather than in cell signaling [6]. Siglec-1-positive macrophages can bind regulatory T cells, negatively controlling their expansion and autoimmune disease progression [25]. Siglec-1-positive macrophages can present lipid antigens to iNKT cells, mediate its early activation in lymph nodes [7]. Siglec-1 can also adhere in a sialic acid dependent manner to various lymphohematopoietic cells [26]. Furthermore, Siglec-1-expressing monocytes can adsorb HIV-1 [9], [27] and porcine reproductive and respiratory syndrome virus (PRRSV) [8] through interaction with the sialic acid residues on the viral envelope glycoprotein. PRRSV is co-localized with its internalization receptor, Siglec-1, on the cell surface and enters its target cell via clathrin-mediated endocytosis [28]. These findings revealed an important role of Siglec-1 in cell adhesion and phagocytosis.

In this study, we found that by the stimulation of oxLDL, many pattern recognition receptors (PRR) on macrophage were up-regulated, including SR-BI, CD64 (FcγRI), CD32B (FcγRIIB), LOX-1, TLR-4, and Siglec-1. Most of these receptors (SR-BI, CD64, CD32B, LOX-1) are well defined oxLDL receptors. LOX-1 is a member of C-type lectins, which can bind to carbohydrate domain in the presence of calcium [29]. LOX-1 was mainly expressed on endothelial cells and firstly identified as a receptor for oxLDL in 1997 [30]. Previous study [17] also found that TLR-4 can recognize minimally oxidized low-density lipoprotein (mmLDL), activate the Vav1-Ras-Raf-MEK-ERK1/2 signaling cascade and enhance uptake of both native and oxidized LDL. These data describe a novel mechanism leading to enhanced lipoprotein uptake in macrophages and suggest that mmLDL is an endogenous ligand for TLR4 [31]. Up-regulation of Siglec-1 by oxLDL stimulation also implies that Siglec-1 may take part in oxLDL internalization on macrophages. More importantly, when Siglec-1 on macrophages was inhibited by siRNA, the uptake of oxLDL by macrophages was significantly diminished. Our findings indicate an important role of Siglec-1 in oxLDL internalization and suggest that oxLDL may be a new ligand for Siglec-1.

It is important to assess the interaction between two pure substances in a system that introduces no other impurities. So we utilized an ELISA-based cell-free system to observe the interaction between Siglec-1 and oxLDL. Unfortunately, no direct interaction between Siglec-1 (human or mouse) and oxLDL (even though desialylated) was observed, as compared with BSA. Furthermore, we also improve the coated Siglec-1 concentration to 10 µg/ml or 20 µg/ml and increase BSA concentration in the assay buffer to exclude non-specific binding, but no interaction was found (data not shown). However, the binding of Siglec-1 to plastic may also prevent its effective receptor function. So we can conclude that Siglec-1 may not interact directly with oxLDL in the *in vitro* ELISA system. Other experiments may be needed to testify Siglec-1-oxLDL interaction in the physiological and pathophysiological conditions. Results also indicated that Siglec-1 may not be an independent receptor for oxLDL, it may act as a co-receptor or cooperate with other oxLDL receptors such as SR in macrophage uptake of oxLDL. So we used LSCM and co-IP to verify our hypothesis.

Recent work has demonstrated that different PRRs can functionally cooperate in macrophage–pathogen interactions and signaling. Seimon et al [32] found that SR-A ligands trigger apoptosis in endoplasmic reticulum-stressed macrophages by cooperating with another PRR, TLR-4, to redirect TLR-4 signaling from prosurvival to proapoptotic. Previous study has shown that co-ligation of another Siglecs member, Siglec-3/CD33, and myeloid activatory receptor CD64 actually reduces activatory signals (calcium mobilization) [33]. Van Gorp et al [34] found that Siglec-1 and another SR member, CD163, joined forces during entry of the PRRSV to macrophages. When cooperated with CD163, Siglec-1 increased virus production 10–100 times and both internalization and uncoating were observed. Our study found that Siglec-1 and SR-BI/CD64 have a co-localization on oxLDL-activated macrophage surface. However, someone may concerns that the co-localization of Siglec-1 with CD64 and SR-BI is not convincing enough, as the LSCM is only a preliminary experiment to find colocalization of two molecules. So we used co-IP to verify the interaction. By the stimulation of oxLDL, LPS or PMA, Siglec-1 and SR-BI can be immunoprecipitated by each other, which indicated that these two receptors can work in a cooperative manner and their interaction may have an important role in oxLDL internalization and signaling on macrophages. Other study found that Siglec-1 internalization was shown to be clathrin- and Eps15-dependent and to result in targeting to early endosomes but not lysosomes [35]. We also exclude the possibility that Siglec-1/SR-BI was immunoprecipitated with the entire caveolae compartment by caveolin-1 detection in the IP products (with no signal).

In summary, oxLDL can stimulate the expression of various PRRs expression on macrophages, including SRs, FCγRs, TLRs and Siglec-1. Target knockdown of Siglec-1 can reduce macrophages uptake of oxLDL. Siglec-1 can interact with SR-BI in the phagocytosis of oxLDL by macrophages, but not act as an independent receptor for oxLDL recognition and internalization. Thus, pharmacological approaches targeting Siglec-1 may prevent oxLDL uptake by macrophages and shed new light on the treatment of AS.

## Supporting Information

Figure S1 **BMMs culture and transfection with siRNA.** (A), On day 6, oxLDL 100 µg/ml were added and incubated for another two days. Short arrows indicated macrophages uptake of oxLDL and turn into foam cells. (B), Cells were transfected with FAM-labeled siRNA and transfection efficiency was evaluated more than 80%. (C–G), 48 h after transfection, cells were collected and QRT-PCR (C), western blot (D,E) and FACS (F, G) were used for Siglec-1 knockdown detection. Data were shown as mean (SD) (n = 6). *p<0.05, **p<0.01, ***p<0.001, vs. mock group.
(TIF)

Figure S2 **Inhibition of Siglec-1 does not affect other major oxLDL receptors.** BMMs were transfected with siRNA targeting Siglec-1, 24 h later oxLDL 100 µg/ml was added to culture medium and incubated for another two days. Then cells were collected and western blot was used to detect SR-BI, CD64, CD36, SR-A and Siglec-1. Na/K ATPase was used as loading control. No major oxLDL receptor was affected by Siglec-1 inhibition except for Siglec-1 itself.
(TIF)

Table S1 **Primary Antibodies Used for Western Blot.**
(DOC)

File S1 (DOC)
