# Supplementary figures and images for: The Role of Siglec-1 and SR-BI Interaction in the Phagocytosis of Oxidized Low Density Lipoprotein by Macrophages
Source: PLoS One. 2013 Mar 8;8(3):e58831. doi: 10.1371/journal.pone.0058831 (PMC3592837; doi:10.1371/journal.pone.0058831)

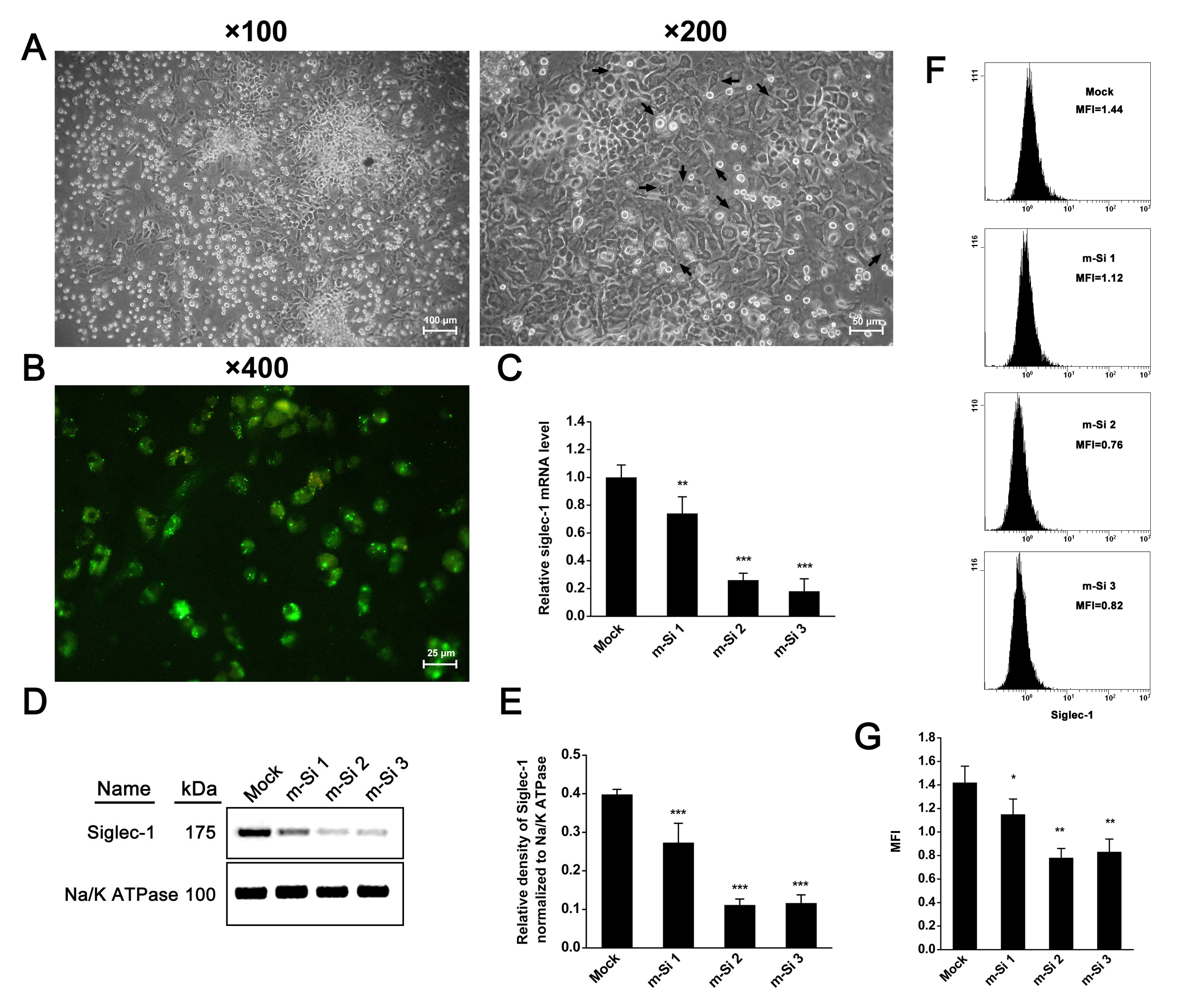

Supplement: Figure S1 — BMMs culture and transfection with siRNA. (A), On day 6, oxLDL 100 µg/ml were added and incubated for another two days. Short arrows indicated macrophages uptake of oxLDL and turn into foam cells. (B), Cells were transfected with FAM-labeled siRNA and transfection efficiency was evaluated more than 80%. (C–G), 48 h after transfection, cells were collected and QRT-PCR (C), western blot (D,E) and FACS (F, G) were used for Siglec-1 knockdown detection. Data were shown as mean (SD) (n = 6). *p<0.05, **p<0.01, ***p<0.001, vs. mock group. (TIF) [file pone.0058831.s001.tif]

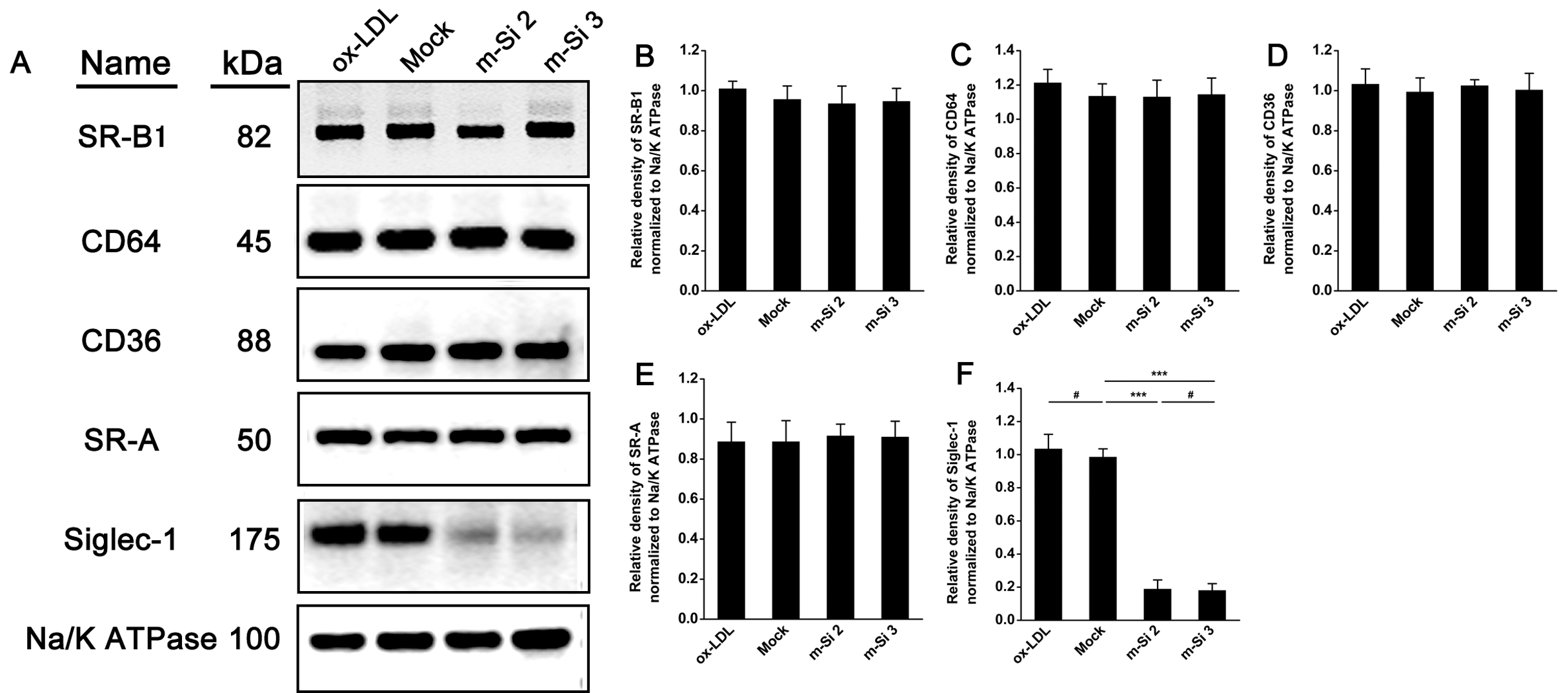

Supplement: Figure S2 — Inhibition of Siglec-1 does not affect other major oxLDL receptors. BMMs were transfected with siRNA targeting Siglec-1, 24 h later oxLDL 100 µg/ml was added to culture medium and incubated for another two days. Then cells were collected and western blot was used to detect SR-BI, CD64, CD36, SR-A and Siglec-1. Na/K ATPase was used as loading control. No major oxLDL receptor was affected by Siglec-1 inhibition except for Siglec-1 itself. (TIF) [file pone.0058831.s002.tif]
