# Supplementary material for: The Role of Siglec-1 and SR-BI Interaction in the Phagocytosis of Oxidized Low Density Lipoprotein by Macrophages
Source: PLoS One. 2013 Mar 8;8(3):e58831. doi: 10.1371/journal.pone.0058831 (PMC3592837; doi:10.1371/journal.pone.0058831)
Supplement: Table S1 — Primary Antibodies Used for Western Blot. (DOC) [file pone.0058831.s003.doc]

**Table. S1. Primary Antibodies Used for Western Blot.**

| **Mouse antigens** | **Poly-/mono-clonal** | **Manufacturer** | **Concentrations (μg/ml) or dilutions** |
| --- | --- | --- | --- |
| Siglec-1 | rat monoclonal | Abcam, Cambridge, UK. | 0.5, 1 |
| Siglce-1 | sheep polyclonal | R&D Systems, Minneapolis, MN. | 0.5, 1, 2 |
| SR-BI | rabbit polyclonal | Thermo Scientific, Rockford, IL | 1:500, 1:1000, 1:2000 |
| SR-BI | rabbit polyclonal | Abcam, Cambridge, UK. | 1, 2 |
| CD64 | rat monoclonal | R&D Systems, Minneapolis, MN. | 0.5, 1, 2 |
| CD32B | goat polyclonal | Santa Cruz Biotechnology, Santa Cruz, CA. | 0.5, 1, 2, 3 |
| TLR-4 | rabbit polyclonal | Abcam, Cambridge, UK. | 0.5, 1, 2, 3 |
| LOX-1 | goat polyclonal | Santa Cruz Biotechnology, Santa Cruz, CA. | 0.5, 1, 2, 3 |
| SRA | rabbit polyclonal | Thermo Scientific, Rockford, IL | 1, 2, 4 |
| CD36 | rat monoclonal | R&D Systems, Minneapolis, MN. | 0.5, 1, 2 |
| caveolin-1 | rabbit polyclonal | Thermo Scientific, Rockford, IL | 0.5, 1, 2, 4 |
| Na/K ATPase | rabbit polyclonal | Cell Signaling, Beverly, MA | 1:500, 1:1000, 1:2000 |
