# Supplementary material for: The Role of Siglec-1 and SR-BI Interaction in the Phagocytosis of Oxidized Low Density Lipoprotein by Macrophages
Source: PLoS One. 2013 Mar 8;8(3):e58831. doi: 10.1371/journal.pone.0058831 (PMC3592837; doi:10.1371/journal.pone.0058831)
Supplement: File S1 — (DOC) [file pone.0058831.s004.doc]

**Detailed Methods**

**Reagents**

Recombinant mouse macrophage colony-stimulating factor (M-CSF), recombinant mouse siglec-1, recombinant human siglec-1, recombinant human CD36, sheep polyclonal anti-mouse Siglec-1 and rat monoclonal anti-mouse Fcγ RI/CD64 were from R&D Systems (Minneapolis, MN). Rabbit polyclonal to SR-A, rabbit polyclonal to SR-BI and rabbit polyclonal to caveolin-1 were from Thermo Scientific (Rockford, IL). Rabbit polyclonal to Na/K ATPase was from Cell Signaling Technology (Beverly, MA). Rat monoclonal (3D6.112) to siglec-1, rabbit polyclonal to scavenging receptor SR-BI, rabbit polyclonal to TLR-4 and rabbit polyclonal to copper oxidized LDL were from Abcam (Cambridge, UK). Goat polyclonal to siglec-1, goat polyclonal to CD32B and goat polyclonal to LOX-1 were from Santa Cruz Biotechnology (Santa Cruz, CA). Rabbit polyclonal to HOCL oxidized LDL was from Chemicon (part of Millipore, Billerica, MA). DyLight™ 488 conjugated donkey anti-goat IgG, DyLight™ 549 conjugated goat anti-rat IgG, DyLight™ 549 conjugated goat anti-rabbit IgG, DyLight™ 488 conjugated goat anti-rabbit IgG, DyLight™ 488 conjugated rabbit anti-sheep IgG, HRP conjugated goat anti-rat IgG, HRP conjugated rabbit anti-sheep IgG, HRP conjugated goat anti-rabbit IgG and HRP conjugated rabbit anti-goat IgG were from Jackson ImmunoResearch (West Grove, PA). Immunoprecipitation kit and BCA protein assay kit were from Pierce (Rockford, IL). Transmembrane protein extraction kit was from Novagen (part of Merck Chemicals, Darmstadt, Germany). Sialidase, PMA, LPS, Oil red O and other chemicals were from Sigma-Aldrich (St. Louis, MO)

**Ox-LDL**

Oxidization of LDL was performed as described previously. Briefly, human LDL was purified from normolipidemic human plasma via density gradient ultra-centrifugation (d = 1.019-1.063 g/ml) and oxidized using 5 μM Cu_2_SO_4_ in PBS at 37°C for 24 hours. Oxidation was terminated by adding excess EDTA. Ox-LDL was analyzed on agarose gel electrophoresis for migration versus LDL. OxLDL migrated 2.5 fold further than the native LDL. Thiobarbituric acid reactive substances (TBARS) for lipid peroxidation was determined colorimetrically by using malondialdehyde as a standard.

**Mouse bone marrow-derived macrophages (BMMs)**

All animals received humane care and protocols for animal experiments were approved by the institutional animal use committee of the Second Military Medical University. BMMs were isolated as described (Cold Spring Harb. Protoc.; 2008; doi:10.1101/pdb.prot5080). Briefly, Six to eight weeks old C57BL/6 mice or BALB/C mice were sacrifice by cervical dislocation and femur and tibia were obtained sterilely. Then the knee joint was cut and the bones were flushed with sterile saline using a 5-ml syringe and a 25-gauge needle. Cells were passed through a strainer (75μm) and resuspended at a concentration of 2×10^6^ cells/ml in complete RPMI-1640 medium (Gibco, Grand Island, NY) containing 10 ng/ml M-CSF. Cells were cultured in 6-well plates (Corning Incorporated, Corning, NY), washed twice with PBS every 2-3 d and fresh medium were added. Six days later, the efficiency of the differentiation was assessed using fluorescence-activated cell sorting (FACS) analysis of Mac-1 and F4/80 surface antigen expression.

**FACS**

Mouse bone marrow-derived macrophages (BMMs) were stimulated with different concentration of ox-LDL (0, 12.5, 25, 50, 100 μg/ml) for 48h and harvested by 0.25% trypsin-1mM EDTA solution (Gibco). FACS was performed as described . Briefly, 2×10^5^ cells in 100 µl staining buffer (PBS + 0.5% BSA + 0.05% sodium azide) were firstly Fc-blocked with 2 μg of mouse IgG for 15 minutes at room temperature and subsequently incubated with antibody for siglec-1, CD64, CD32B, TLR-4, LOX-1 or SR-BI at a concentration of 10 μg/ml for 1 h. After wash, cells were resuspended in 100 µl staining buffer, stained with appropriate DyLight™ conjugated secondary antibody (Double staining: DyLight™ 488 rabbit anti-sheep for Siglec-1 and then DyLight™ 549 goat anti-rat for CD64, Sheep IgG (2 μg) was used between steps to block the free valencies of the secondary antibody; DyLight™ 488 donkey anti-goat for CD32B & LOX-1 and then DyLight™ 549 goat anti-rat for Siglec-1, Goat IgG (2 μg) was used between steps to block the free valencies of the secondary antibody; DyLight™ 549 goat anti-rat for Siglec-1 and then DyLight™ 488 goat anti-rabbit for SR-BI & TLR-4.) at a concentration of 5 μg/ml for 30 min. And then washed and resuspended in 500 μl PBS. Cells were analyzed by FC500 flow cytometer (Beckman Coulter, Fullerton, CA) and CXP Analysis Softwares (Beckman Coulter). Appropriate isotype-matched control antibodies were used in parallel.

**Cell transfection and RNA interference**

BMMs at 50–70% confluence were transfected with siRNA (40 nmmol/L) pre-mixed with lipofectamine 2000 (Invitrogen, Carlsbad, CA) (1:2, μg: μl) in antibiotics-free serum-free DMEM culture medium according to the manufacturer’s instructions. SiRNA corresponding to a 21-nucleotide sequence targeting 2176–2196 (m-si 1), 3233-3253 (m-si 2), and 4625-4645 (m-si 3) of mouse siglec-1 (**NM_011426.3**), i.e. 5’- UUAGCAUAGAAGUCUAGGCTG-3’, 5’- UAGUUCUCGGACCUCUAGCTG-3’ and 5’- UUGUGUAUCAUGCACCUGGCA-3’ were synthesized by GenePharma (Shanghai, China). A fluorescein conjugated non-targeting siRNA was used to monitor the transfection efficiency in parallel. Six hours after transfection, culture medium was changed into complete DMEM culture medium with antibiotics and FBS. Eighteen hours after transfection, transfection efficiency was determined by fluorescence microscopy. Forty-eight hours after transfection, the expression of target gene was further examined by quantitative RT-PCR and western blot.

**Semi-quantitative RT-PCR**

PCR analysis was performed as described previously . Briefly, total RNA was extracted by using RNeasy mini kit (Qiagen, Hilden, Germany). To avoid genomic DNA contamination, DNA degradation was performed by using RQ1 RNase-Free DNase (Promega, Madison, WI). cDNA was synthesized by using the SuperScript III First-Strand Synthesis kit (Invitrogen) with oligo dT primers. Primers were designed with the Primer Express software, version 3.0 (Applied Biosystems, Foster City, CA) and verified to generate a single product specific to target genes by BLAST algorithm (http://www.ncbi.nlm.nih.gov/blast/). Primers were as follow: mouse Siglec-1 (**NM_011426.3**), sense-primer, 5’-CCTGGTGTGCAGTGTACAAAGTG-3′, antisense-primer, 5’-CCGCGCCTTGTAGGGTAGA-3’, amplicon size 89 bp; mouse GAPDH (**NM_008084.2**), sense-primer, 5’-TGGCCTCCAAGGAGTAAGAAAC-3’, antisense-primer, 5’-GGGATAGGGCCTCTCTTGCT-3’, amplicon size 72 bp. Real-time PCR reactions were performed by using the ABI 7000 System with SYBR® Green PCR Master Mix (Applied Biosystems). Specificity of the products was confirmed by melting curve analysis and gel electrophoresis. As a control for cross contamination samples consisting of distilled water were also subjected to the isolation procedures and the extracts were tested with all assays. Cycle Threshold (Ct) values were calculated after confirming similar amplification efficiencies of target gene and endogenous control. Results were analyzed using ∆∆Ct method .

**Oil red O staining**

BMMs were plated on chamber slides and stimulated with ox-LDL 100 μg/ml for 48h. After incubation, the cells were washed with PBS, fixed with 4% paraformaldehyde for 10 minutes, and stained with a saturated concentration of oil red O in 60% isopropanol for 1h at 55°C. After washed with PBS, slides were mounted with glycerogelatin and photographed with a Leica DM LB microscope (Leica Microsystems, Wetzlar, Germany) equipped with an Olympus DP70 CCD camera (Olympus Optical Co., Ltd, Japan). For quantitation of lipid accumulation in cells, 1×10^5^ cells were washed, fixed and stained as above. And then cells were washed with 60% isopropanol for 5 s and incorporated stain was eluted with 300 μl 60% isopropanol and the optical density (OD) of the solution at 510 nm was measured .

**Siglec-1-ox-LDL interaction assay by ELISA**

ELISA for protein interaction was performed as described previously . Briefly, recombinant human or mouse siglec-1 (0.2 μg~0.5 μg /100 μl, 5197-SL, 5610-SL, both from R&D systems) or BSA (0.2 μg /100 μl, as a negative control) was immobilized to each well of 96-well ELISA plates (high-binding, Corning Costar 9018) by incubation overnight (16-20h) at 4 °C in PBS. After 2 washes with wash buffer (PBS+0.1% Tween 20), the plates were blocked with 300 μl blocking buffer (3% BSA/PBS) at 4 °C for 12 h. After 2 washes with wash buffer, different concentration of ox-LDL (1.53 ng/ml~200 μg/ml) in the 1×Assay Diluent (eBioscience, San Diego, CA) was added to each well, and incubated at 4 °C overnight. In some cases, *Vibrio cholerae* sialidase (50 mU/ml, Sigma) was used to treat rh-Siglec-1 and oxLDL for 1 hour at 37°C before adding them to the well . The plates were then washed and incubated with rabbit polyclonal to ox-LDL (1:1000, Abcam or Chemicon) in Assay Diluent at 4 °C overnight. After wash, plates were incubated with HRP conjugated goat anti-rabbit (1:5000, Jackson ImmunoResearch) in Assay Diluent for 2h at room temperature. After 7 washes with wash buffer, peroxidase activity was determined with a TMB Substrate Solution (eBioscience) and absorbance at 450 nm was measured on an automatic plate reader (Bio-Rad Laboratories, Hercules, CA). For standard curve, 2-fold serial diluted ox-LDL (1.53 ng/ml~200 μg/ml) was immobilized directly to 96-well ELISA plates and blocked, subsequently primary and secondary antibody were used and peroxidase activity was determined as above. For negative control, the primary antibody was substituted with normal rabbit serum at the same dilution. A known oxLDL receptor CD36 was used as positive control to validate the ELISA system.

**Laser Scanning Confocal Microscopy (LSCM)**

BMMs were plated on 6-well plate with sterile coverslip, stimulated with ox-LDL 100 μg/ml for 48h. Culture medium was discarded and cells were washed with PBS twice and fixed with 4% paraformaldehyde for 10 minutes. After Fc-blocked with blocking buffer (PBS + 0.5% BSA + 10%FBS) for 40 min, cells were incubated simultaneously with anti-siglec-1 and one of the following antibody: CD36, SR-A, CD64, CD32B, TLR-4, LOX-1 or SR-BI at a concentration of 20 μg/ml diluted in blocking buffer overnight at 4°C in a humidified container. Appropriate isotype-matched IgG or normal serum was used in parallel as control. After washed with PBS, cells were incubated successively with appropriate DyLight™ conjugated secondary antibody (DyLight™ 488 rabbit anti-sheep for Siglec-1 and then DyLight™ 549 goat anti-rat for CD64 & CD36, Sheep IgG (2 μg) was used between steps to block the free valencies of the secondary antibody; DyLight™ 488 donkey anti-goat for CD32B & LOX-1 and then DyLight™ 549 goat anti-rat for Siglec-1, Goat IgG (2 μg) was used between steps to block the free valencies of the secondary antibody; DyLight™ 549 goat anti-rat for Siglec-1 and then DyLight™ 488 goat anti-rabbit for SR-A, SR-BI & TLR-4.) at a concentration of 10 μg/ml diluted in blocking buffer for 30 min in the dark, with wash steps between each incubation. Then coverslips were mounted with Prolong® Gold Antifade Reagent (Invitrogen) and analyzed using a Leica TCS SP2 confocal microscope and TCSNTV software (Leica Microsystems).

**Co-immunoprecipitation (Co-IP) and Immunoblotting (IB)**

BMMs were stimulated with ox-LDL (100 μg/ml), PMA(20ng/ml) or LPS(0.1μg/ml) for 48h, washed 3 times with ice-cold PBS and lyzed and cell membrane protein was extracted by using transmembrane protein extraction kit (Novagen) with Extraction Buffer 2A. Protein concentrations were determined using the BCA protein assay kit (Pierce). A Pierce® Classic IP Kit was used for Co-IP. Briefly, 3 μg of rat anti-siglec-1 antibody (3D6.112) or rabbit polyclonal to SR-BI (Thermo Scientific) was mixed with 0.5 mg extracted membrane protein in a microcentrifuge tube overnight at 4ºC to form immune complex. Then the immune complex were captured and eluted according to the manufacturer's instructions.

Twenty micrograms per lane of immunoprecipitated protein were mixed with one-fifth volume of sample buffer and boiled for 5 min before being loaded onto a 5% stacking/12% resolving gel (5% stacking/8% resolving gel for Siglec-1) for electrophoresis. Separated proteins were transferred to PVDF membrane (Millipore, Billerica, MA) using an Mini Trans-Blot® Electrophoretic Transfer Cell (Bio-Rad) with transfer buffer (25 mmol/L Tris base, 192 mmol/L glycine, 20% methanol, PH 8.3) at 300 mA for 1h in ice bath. Blots were rinsed in water, washed in TBST (20 mmol/L Tris-HCl (pH 7.6)/137 mmol/L NaCl/0.1% Tween 20) and incubated 1.5 h in blocking solution (5% w/v nonfat dry milk in TBST). Blots were incubated overnight at 4°C in blocking solution containing primary antibodies for Siglec-1, CD36, SR-A, CD64, CD32B, TLR-4, SR-BI, LOX-1, caveolin-1 or Na/K ATPase (concentrations between 0.5~4 μg/ml, see Table. s1). The next day, membranes were washed 5 times, 5 min each, in TBST prior to 1h incubation at room temperature with appropriate HRP-conjugated secondary antibody (Jackson ImmunoResearch, 1:5000~1:10000 dilution, goat anti-rat for CD64 and CD36; rabbit anti-sheep for Siglec-1; goat anti-rabbit for SR-A, SR-B, TLR-4, caveolin-1 and Na/K ATPase; and rabbit anti-goat for CD32B and LOX-1) diluted in blocking solution. After membranes were washed for 7 times, 5 min each in TBST, signal was detected by using ECL chemiluminescent substrate (Millipore) and recorded by X-ray films (Kodak, Rochester, NY).

**Statistical analysis**

Data were shown as mean±SD. Statistical analysis was performed using the SPSS V.15.0 for Windows software (SPSS Inc., Chicago, IL). Normally distributed data were analyzed using one-way ANOVA followed by Student-Newman-Keuls post-hoc test. For non-normally distributed data, significant analyses between more than two groups was performed using Kruskal–Wallis H test instead, significant difference between two groups was analyzed using Mann–Whitney U test. The significance level was set at *P* < 0.05.

**References**

1. Kunjathoor VV, Febbraio M, Podrez EA, Moore KJ, Andersson L, et al. (2002) Scavenger receptors class A-I/II and CD36 are the principal receptors responsible for the uptake of modified low density lipoprotein leading to lipid loading in macrophages. J Biol Chem 277: 49982-49988.

2. Zhang J, Silva T, Yarovinsky T, Manes TD, Tavakoli S, et al. (2010) VEGF blockade inhibits lymphocyte recruitment and ameliorates immune-mediated vascular remodeling. Circ Res 107: 408-417.

3. Levy AP, Purushothaman KR, Levy NS, Purushothaman M, Strauss M, et al. (2007) Downregulation of the hemoglobin scavenger receptor in individuals with diabetes and the Hp 2-2 genotype: implications for the response to intraplaque hemorrhage and plaque vulnerability. Circ Res 101: 106-110.

4. Xiong YS, Zhou YH, Rong GH, Wu WL, Liang Y, et al. (2009) Siglec-1 on monocytes is a potential risk marker for monitoring disease severity in coronary artery disease. Clin Biochem 42: 1057-1063.

5. Livak KJ, Schmittgen TD (2001) Analysis of relative gene expression data using real-time quantitative PCR and the 2(-Delta Delta C(T)) Method. Methods 25: 402-408.

6. Janke J, Engeli S, Gorzelniak K, Luft FC, Sharma AM (2002) Mature adipocytes inhibit in vitro differentiation of human preadipocytes via angiotensin type 1 receptors. Diabetes 51: 1699-1707.

7. Ramirez-Zacarias JL, Castro-Munozledo F, Kuri-Harcuch W (1992) Quantitation of adipose conversion and triglycerides by staining intracytoplasmic lipids with Oil red O. Histochemistry 97: 493-497.

8. Fujita Y, Kakino A, Nishimichi N, Yamaguchi S, Sato Y, et al. (2009) Oxidized LDL receptor LOX-1 binds to C-reactive protein and mediates its vascular effects. Clin Chem 55: 285-294.

9. Kivi E, Elima K, Aalto K, Nymalm Y, Auvinen K, et al. (2009) Human Siglec-10 can bind to vascular adhesion protein-1 and serves as its substrate. Blood 114: 5385-5392.

10. Barnes YC, Skelton TP, Stamenkovic I, Sgroi DC (1999) Sialylation of the sialic acid binding lectin sialoadhesin regulates its ability to mediate cell adhesion. Blood 93: 1245-1252.
